# Efficacy of poly (ADP-ribose) polymerase inhibitors monotherapy and the impact to subsequent platinum-based chemotherapy in breast cancer susceptibility genes1/2-mutated ovarian cancer patients with secondary platinum-sensitive relapse

**DOI:** 10.1186/s13048-023-01283-2

**Published:** 2023-10-28

**Authors:** Yana Ma, Jiale Liu, Ning Li, Hualei Bu, Yongwen Huang, Chengjuan Jin, Hao Wen, Shuai Feng, Hui Zhang, Xiaorong Yang, Beihua Kong, Lingying Wu, Kun Song

**Affiliations:** 1 Department of Obstetrics and Gynecology, Qilu Hospital of Shandong University, 107 Wenhua Xi Road, Jinan, 250012 Shandong Province China; 2 Gynecologic Oncology Key Laboratory of Shandong Province, Qilu Hospital of Shandong University, Jinan, 250012 China; 3 Department of Gynecologic Oncology, National Cancer Center/National Clinical Research Center for Cancer/Cancer Hospital, Chinese Academy of Medical Sciences and Peking Union Medical College, No. 17 Panjiayuan Nanli, Beijing, 100021 Chaoyang District China; 4 Gynecologic Department, Sun Yat-sen University Cancer Center, Guangzhou, 510060 China; 5 Department of Obstetrics and Gynecology, School of Medicine, Shanghai General Hospital, Shanghai Jiao Tong University, Shanghai, 201620 China; 6 Department of Gynecologic Oncology, Fudan University Shanghai Cancer Center, Shanghai, 201620 China; 7 Gynecological Oncology Department, Shandong Cancer Hospital and Institute, Shandong First Medical University and Shandong Academy of Medical Sciences, Jinan, 250012 China; 8 Department of Gynecology, The Fourth Hospital of Hebei Medical University, Shijiazhuang, 050000 China; 9 Clinical Epidemiology Unit, Qilu Hospital of Shandong University, Jinan, 250012 China

**Keywords:** PARPi monotherapy, Platinum-sensitive recurrence, BRCA1/2 mutation, Post-recurrence survival

## Abstract

**Background:**

The therapeutic effect of poly (ADP-ribose) polymerase inhibitors (PARPi) monotherapy compared with platinum-based chemotherapy, and the impact to subsequent platinum-based chemotherapy after PARPi resistance were inconclusive in breast cancer susceptibility genes (BRCA)1/2-mutated ovarian cancer patients with secondary platinum-sensitive relapse.

**Methods:**

BRCA1/2-mutated patients with secondary platinum-sensitive relapse included in this study did not receive any maintenance regimen after first- and second-line platinum-based chemotherapy, and the secondary platinum-free interval (PFI) was more than 6 months. Patients in study group were treated with PARPi monotherapy until disease progression, and patients in control group were treated with platinum-based chemotherapy without restriction. Progression-free survival (PFS) was defined as the time from third-line therapy to disease progression or death, PFS2 was defined as the time from platinum-based chemotherapy after PARPi resistance to next subsequent therapy or death. Post-recurrence survival (PRS) refers to the survival time after secondary platinum-sensitive relapse.

**Results:**

A total of 119 patients were retrospectively analyzed, including 71 (59.7%) in study group and 48 (40.3%) in control group. The objective response rate (ORR: 77.5% vs. 80.0%, *p=*0.766) and PFS (median: 11.2 vs. 11.0 months, *p=*0.962) were comparable. The benefit of subsequent platinum-based chemotherapy after PARPi resistance was more pronounced in patients with PARPi treatment for more than 12 months (median PFS2: 8.6 vs. 4.3 months, *p=*0.040). PARPi monotherapy had no adverse effect on PRS compared with platinum-based chemotherapy (median PRS:41.2 vs. 42.8 months, *p=*0.323). Compared to patients in control group who had never received PARPi, PARPi monotherapy (median PRS: 41.2 vs. 33.7 months, *p=*0.019) and post-line treatment with PARPi in the control group (median PRS: 48.1 vs. 33.7 months, *p=*0.002) could prolong PRS for patients with secondary platinum-sensitive relapse.

**Conclusions:**

PARPi monotherapy was similar to platinum-based chemotherapy for BRCA1/2-mutated ovarian cancer patients with secondary platinum-sensitive recurrence, and could improve prognosis.

**Supplementary Information:**

The online version contains supplementary material available at 10.1186/s13048-023-01283-2.

## Background

Ovarian cancer is a common malignant tumor of the female reproductive system and its mortality ranks first among gynecological malignant tumors [1]. Approximately 90% of ovarian cancer are of an epithelial cell type, and the remaining 10% are non-epithelial ovarian cancers, which include mainly germ cell tumours, sex cord-stromal tumours, and some extremely rare tumours such as small cell carcinomas [2]. More than 75% of patients eventually relapse within two years of initial treatment [3], and patients with platinum-sensitive relapse are recommended re-challenge with platinum-based chemotherapy until platinum resistance. However, once platinum resistance occurs, the response rate of subsequent therapies is only about 10-25%, and the prognosis is extremely poor, with median overall survival of only 12 months [3, 4].

BRCA1/2 germline mutations are the strongest known genetic risk factors for epithelial ovarian cancer and are found in 6–16% of patients [5, 6], and treatment with poly (ADP-ribose) polymerase inhibitors (PARPi) has a significant response based on “synergistic lethal effects” [7] . Several PARP inhibitors are currently available for the clinical treatment of patients with ovarian cancer [8], in addition, some studies have compared the differences between PARPi monotherapy and platinum-based chemotherapy. For example, in NGR-GY004 study, the median PFS of PARPi monotherapy for platinum-sensitive recurrent ovarian cancer patients with BRCA1/2 mutations was better than platinum-based chemotherapy, and the ongoing OPAL-C trial (NCT03574779) is exploring the superiority of PARPi monotherapy compared to Platinum-Taxane in neoadjuvant chemotherapy for patients with homologous recombination deficiency (HRD)-positive tumors, however, these studies have not yet published the results of overall survival. Recently, the FDA has withdrawn several indications for PARPi monotherapy due to an increased risk of death in patients with more than three lines of chemotherapy [9, 10], so further clarification is needed as to whether patients with prior 2 lines of chemotherapy would have a survival benefit with PARPi monotherapy

Platinum-free interval (PFI) can be used to predict subsequent chemotherapy response and prognosis [11], and prolongation of PFI (using non-platinum-based regimens) might restore platinum sensitivity and improve survival [12]. However, the PARPi regimen is continuous until disease progression or intolerable toxicity [13, 14], so the concept of PFI has become controversial. As a non-platinum-based regimen, PARPi monotherapy after relapse could prolong the PFI, but it was unknown whether platinum-based chemotherapy was still effective after PARPi resistance, and whether it could prolong the survival of patients .Therefore, we conducted this retrospective analysis to try to address these clinically urgent questions.

## Methods

### Patients and clinical data

**Fig. 1:**
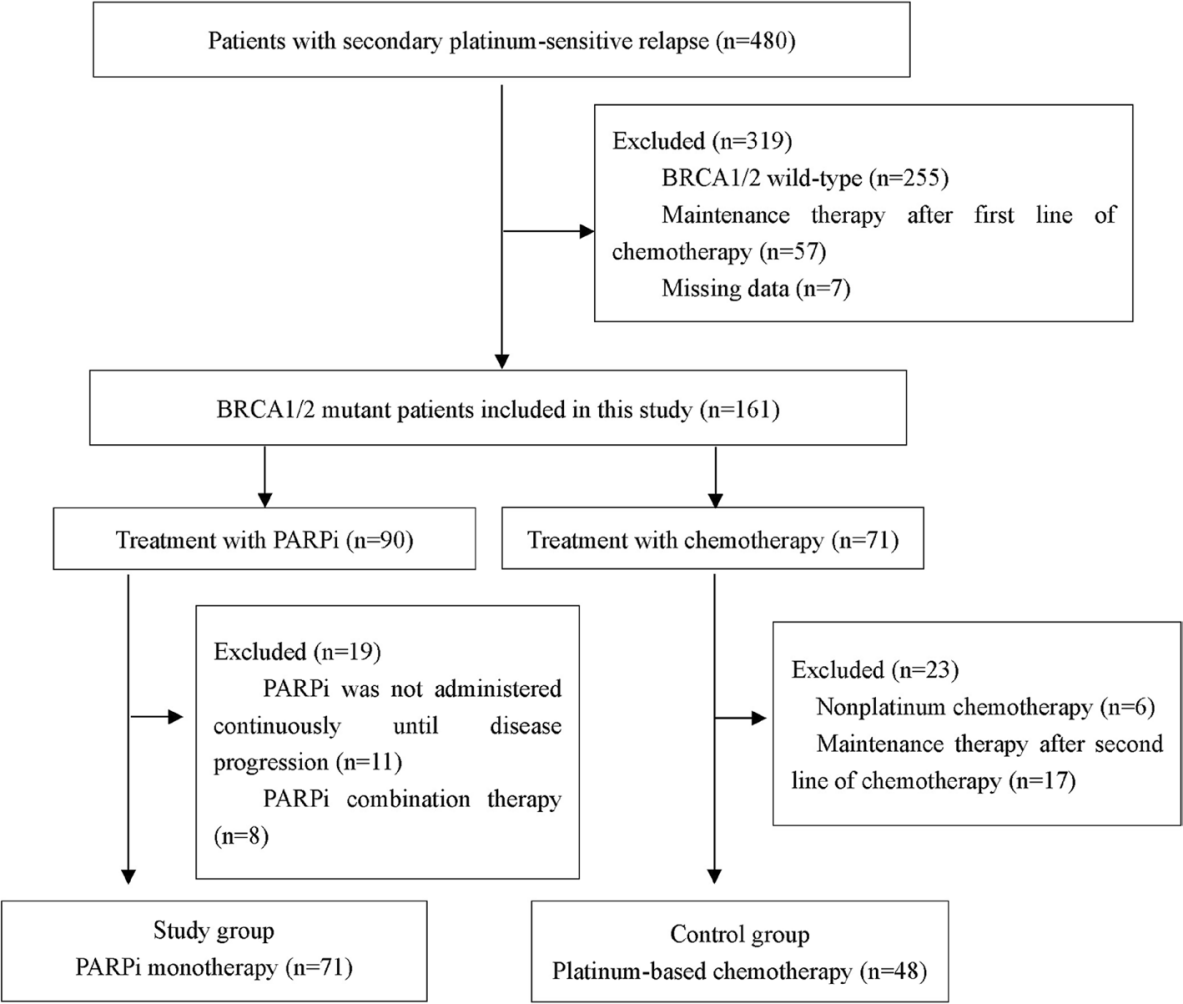
Flow chart of the study population. BRCA, breast cancer susceptibility gene; PARP, poly (ADP-ribose) polymerase (PARP) inhibitors

The flow chart of the study population was shown in Fig.  1. Patients included in this study were diagnosed in Qilu Hospital of Shandong University, Cancer Hospital of Beijing Academy of Medical Sciences, Cancer Hospital affiliated to Sun Yat-sen University, Shanghai General Hospital, Fudan University Shanghai Cancer Center, Shandong Cancer Hospital, and the Fourth Hospital of Hebei Medical University from 2010/02/01 to 2018/09/24, and all carried a germline BRCA1/2 pathogenic mutation. The patient did not receive any maintenance regimen, such as PARPi, bevacizumab, etc., after first- and second-line platinum therapy. The secondary PFI was more than 6 months in all patients. Patients enrolled in the study group were treated with PARPi monotherapy (Fluzoparib, Olaparib and Pamiparib) after secondary platinum-sensitive relapse and continued treatment until disease progression, demonstrating resistance to PARPi. Patients in control group were treated with platinum-based chemotherapy after secondary platinum-sensitive relapse, without restriction on the specific type and dose of platinum. Determination of response to PARPi and platinum-based chemotherapy was performed according to the Response Evaluation of Response in Solid Tumours (RECIST) 1.1 criteria. If clinical data were insufficient for evaluation according to RECIST criteria, the GCIG CA125 criteria were used as an alternative [15].

PFS was defined as time from third-line therapy (including PARPi monotherapy and platinum-based chemotherapy) to disease progression or death, PFS2 was defined as time from platinum-based chemotherapy after PARPi resistance to next subsequent therapy or death. Post-recurrence survival (PRS) refers to the survival time after secondary platinum-sensitive relapse. Additional clinical data were collected including age at diagnosis, primary tumor location, CA-125 level at secondary relapse, neoadjuvant chemotherapy, pathological type, BRCA1/2 germline mutational status, platinum-based chemotherapy regimens, PARPi duration, efficacy and toxicity, primary and secondary PFI, and survival.

### Germline BRCA1/2 detection

The NGS-based BRCA1/2 germline detection protocol included the following six steps: labelling, sample acquisition and processing, nucleic acid extraction, library construction, sequencing, data analysis and mutation interpretation, with corresponding quality controls at each step. For amplicon based and hybridization capture methods, the detection regions included the entire exon coding region of BRCA1/2 gene and the exon-intron interface region (±20 base pairs). The average depth of each run was over X200. Sanger DNA sequencing was performed for all reported variations using specific gene primers. All point mutations and small indels were confirmed by sanger DNA sequencing using specific gene primers, and the large fragment rearrangements were detected by multiplex ligation-dependent probe amplification (MLPA) methods. Variants were named according to HGVS nomenclature, and guidelines for the interpretation of sequence variants into 5-class system adapted from the International Agency for Research on Cancer [16, 17].

## Statistical analyses

Student’s t-test was used to compare differences in continuous variables with normal distribution. Differences in clinical characteristics and post- recurrence survival between defined groups of patients were assessed using chi-square test and Kaplan–Meier methods, where appropriate, and hazard ratios (HR) with 95% confidence intervals (CI) were calculated. In univariate analysis, *p* value 0.10 was defined as the upper limit for inclusion in multivariate analysis, in the latter, *p<*0.05 was considered significant. The SPSS program (version 16.0) was used for all statistical analysis. The significance levels were * *p <* 0.05 and ** *p <* 0.01, respectively.

## Results

### PARPi monotherapy was comparable to platinum-based chemotherapy in patients with secondary platinum-sensitive relapse

A total of 119 patients were eligible for this retrospective analysis, including 71 (59.7%) in study group and 48 (40.3%) in control group. The median age at diagnosis of enrolled patients was 49 years, and the majority (77.3%) of patients had BRCA1 mutations. The baseline characteristics of patients were shown in Table 1 and were generally well balanced in age of diagnosis (*p=*0.446), neoadjuvant chemotherapy followed by interval debulking surgery (NAC-IDS, *p=*0.882), the International Federation of Gynecology and Obstetrics (FIGO) stage (*p=*0.321), residual lesions of primary surgery (*p=*0.770), CA-125 level at secondary platinum-sensitive relapse (*p=*0.677), PFI after 1^st^ (*p=*0.771) and 2^nd^ (*p=*0.141) platinum-based chemotherapy.

**Table 1:** Baseline characteristics of patients with secondary platinum-sensitive relapse

	**Study Group** **(*n =* 71)**	**Control Group** **(*n =* 48)**	***p*-value**
Age at diagnosed (years)			
≤49 years	42 (59.2%)	25(52.1%)	0.446
>49 years	29 (40.8%)	23(47.9%)	
BRCA-germline-mutation status			
BRCA1 mutation	54 (76.1%)	38 (79.2%)	0.691
BRCA2 mutation	17 (23.9%)	10 (20.8%)	
NAC-IDS			
Yes	14 (19.7%)	10 (20.8%)	0.882
No	57 (80.3%)	38 (79.2%)	
FIGO stage at diagnosed			
I/II	12 (16.9%)	5 (10.4%)	0.321
III/IV	59 (83.1%)	43 (89.6%)	
Primary tumor location			
Ovary	69 (97.2%)	47 (97.9%)	0.802
Fallopian tube	2 (2.8%)	1 (2.1%)	
Histologic type			
High-grade serous	68 (95.8%)	44(91.7%)	0.483
Serous not specified	1 (1.4%)	2 (4.2%)	
Endometrioid	2 (2.8%)	1 (0.0%)	
Clear-cell	0 (0.0%)	1 (2.1%)	
Residual lesions			
No	31 (43.7%)	19 (39.6%)	0.770
Yes	20 (28.2%)	14 (29.2%)	
Unknown^a^	20 (28.2%)	15 (31.3%)	
PFI after 1^st^ line of platinum-containing chemotherapy			
<12 months	24 (33.8%)	15 (31.3%)	0.771
≥12 months	47 (66.2%)	33 (68.8%)	
PFI after 2^nd^ line of platinum-containing chemotherapy			
≥6, <12 months	52 (73.2%)	29 (60.4%)	0.141
≥12 months	19 (26.8%)	19 (39.6%)	
CA-125 level at secondary platinum-sensitive relapse			
≤70 U/ml	18 (25.4%)	8 (16.7%)	0.677
>70 U/ml	50 (70.4%)	18 (37.5%)	
Unknown^a^	3 (4.2%)	22 (45.8%)	
Tumor response of 3^rd^ line			
PR/CR	55 (77.5%)	28 (58.3%)	0.766
SD/PD	16 (22.5%)	7 (14.6%)	
Unknown^a^	0 (0.0%)	13 (27.1%)	
Duration of PARPi treatment			
<6 months	7 (9.9%)	--	--
≥6, <12 months	32 (45.1%)	--	
≥12 months	32 (45.1%)	--	
Hematological toxicity (≥ 3 CTCAE)			
Yes	31 (43.7%)	--	--
No	36 (50.7%)	--	
Unknown^a^	4 (5.6%)	--	
Chemotherapy regimens of 3^rd^ line			
Carboplatin based	--	16 (33.3%)	--
Nedaplatin based	--	10 (20.8%)	
Cisplatin based	--	5 (10.4%)	
Oxaliplatin based	--	1 (2.1%)	
Lobaplatin based	--	7 (14.6%)	
Multiple platinum	--	9^b^ (18.8%)	

*BRCA* Breast cancer susceptibility gene, *NAC-IDS* Neoadjuvant chemotherapy and interval debulking surgery, *FIGO* International Federation of Gynecology and Obstetrics, *PFI* Platinum-free interval, *CA* Carbohydrate antigen, *PARP* Poly (ADP-ribose) polymerase (PARP) inhibitors, *CTCAE* Common Terminology Criteria for Adverse Events

^a^Data identified as unknown were not included in the difference analysis between the two groups

^b^Carboplatin+oxaliplatin: 2 patients; Carboplatin+cisplatin: 2 patients; Carboplatin+nedaplatin: 1 patient; Cisplatin+nedaplatin/lobaplatin/oxaliplatin: 3 patients; Lobaplatin+nedaplatin: 1 patient

**Fig. 2:**
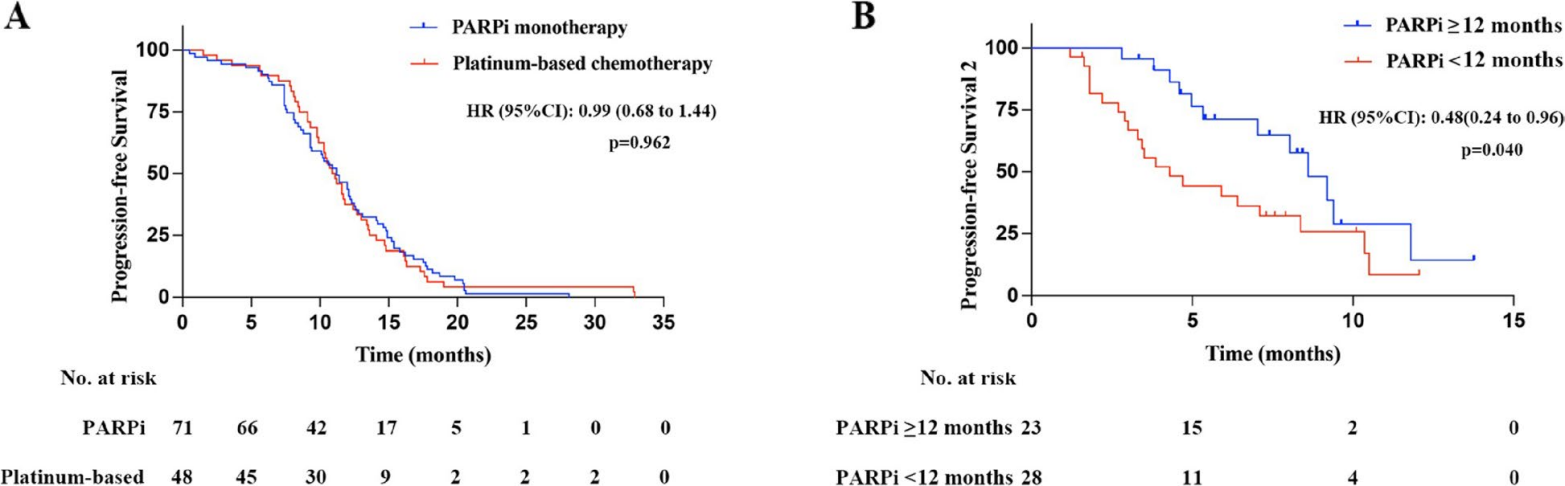
Progression-free survival analysis of patients. **A** PFS of PARPi monotherapy and platinum-based chemotherapy in patients with secondary platinum-sensitive relapse was similar. **B** The benifit of platinum-based chemotherapy after PARPi resistance was positively correlated with the duration of PARPi administration

Tumor response of PARPi monotherapy and platinum-based chemotherapy was the primary observation of this study. Sixty-four patients (90.1%) with PARPi monotherapy were on medication for more than 6 months, and 32 patients (45.1%) for more than 12 months. All 71 patients in study group were evaluable and showed excellent outcomes, with 55 patients (77.5%) meeting the criteria for disease remission. Tumor evaluation was available in 35 patients (72.9%) who received platinum-based chemotherapy, and disease remission was achieved in 28 patients (80.0%), which was comparable to PARPi monotherapy (*p=*0.776), and there was no statistical difference in PFS between the two groups (Fig.  2A; median PFS, 11.2 vs. 11.0 months, HR=0.99, *p=*0.962).

Due to the limitations of retrospective studies, we only collected hematological toxicity of PARPi monotherapy. There were 31(43.7%) patients with grade 3 or higher (CTCAE standard) adverse events related to PARPi, and no patients discontinued medication due to adverse events. Myelodysplastic syndromes and acute myelocytic leukemia did not occur in patients treated with PARPi monotherapy at follow-up.

### The benefit of platinum-based chemotherapy after PARPi resistance was positively correlated with the duration of PARPi administration

Fifty-one (71.8%) patients in study group received platinum-based chemotherapy after PARPi resistance with a median PFS2 of 7.0 months. According to the duration of PARPi treatment, the patients were divided into two groups, including 23 (45.1%) patients with more than 12 months and 28 (54.9%) patients with less than 12 months. The baseline characteristics of patients were shown in Additional file  1, and were generally well balanced. The results showed that the benefit of subsequent platinum-based chemotherapy was more pronounced on patients with PARPi treatment for more than 12 months (Fig. 2B; median PFS2: 8.6 vs. 4.3 months, *p=*0.040).

### PARPi monotherapy after secondary platinum-sensitive relapse had no adverse effect on PRS and could prolong survival time compared to patients without PARPi history

**Fig. 3:**
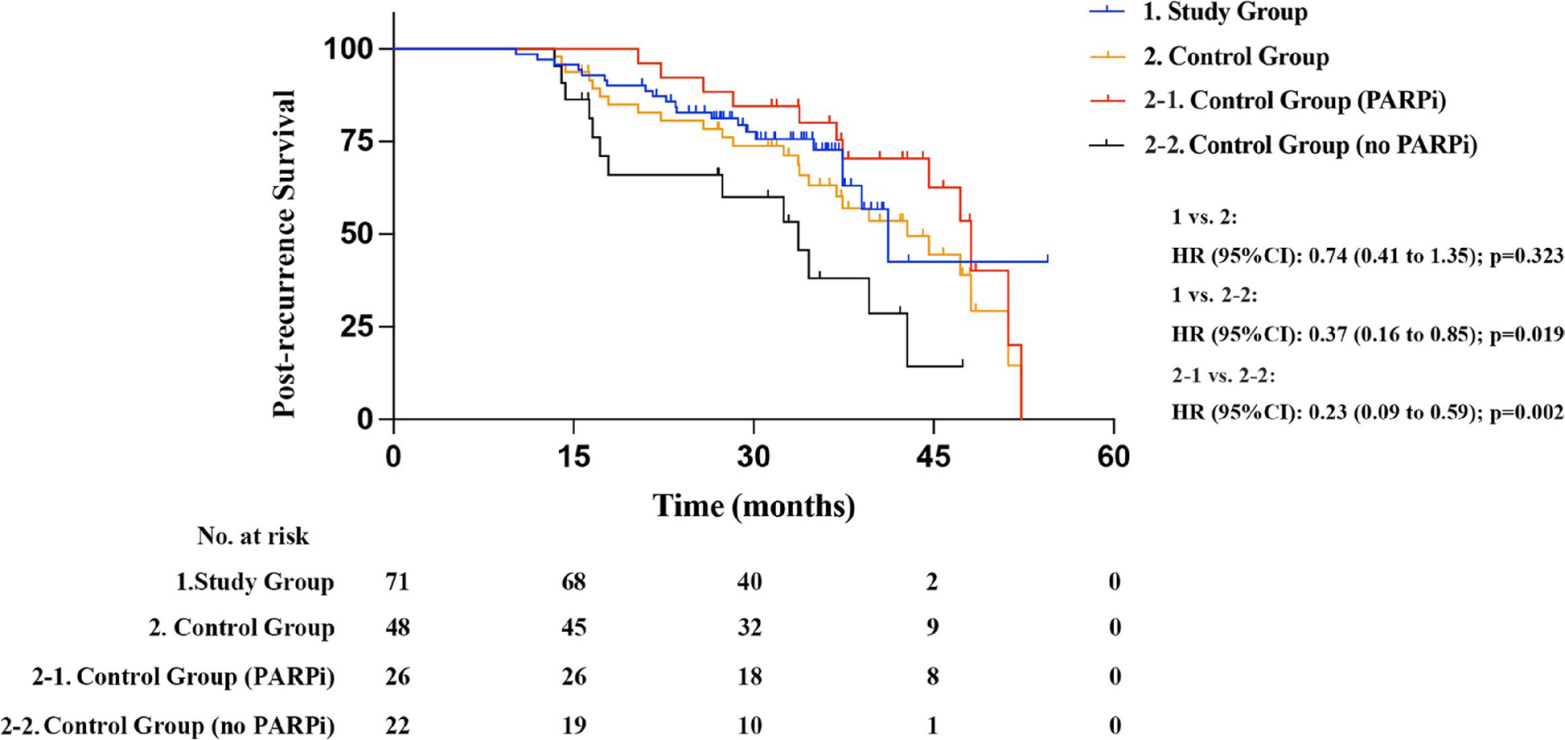
Post-recurrence survival analysis of patients. PARPi monotherapy after secondary platinum-sensitive relapse had no adverse effect on PRS compared with platinum-based chemotherapy; Both PARPi monotherapy after secondary platinum-sensitive relapse and post-line treatment with PARPi prolonged PRS in patients compared to those without PARPi history

The median duration of post-recurrence follow-up for survival analysis was 31.0 months in PARPi monotherapy group vs. 34.2 months in control group. PARPi monotherapy after secondary platinum-sensitive relapse had no adverse effect on PRS compared with platinum-based chemotherapy (Fig. 3, median PRS: 41.2 vs. 42.8 months, *p=*0.323). Of note, 26 patients in control group were treated with PARPi in subsequent therapy and subgroup analysis was performed, the results showed that both PARPi monotherapy after secondary platinum-sensitive relapse (Fig.  3, median PRS: 41.2 vs. 33.7 months, *p=*0.019) and post-line treatment with PARPi (Fig.  3, median PRS: 48.1 vs. 33.7 months, *p=*0.002) prolonged PRS in patients compared to those who had never used PARPi.

Univariate analysis was performed, and included the following variables: age at diagnosis, BRCA1/2 mutation status, PARPi monotherapy after secondary platinum-sensitive relapse or not, PARPi history in post-line treatment, residual lesions (R0, no residual lesions; R1, residual lesions less than 1 cm; R2, residual lesions more than 1 cm), FIGO stage, NAC-IDS, CA-125 level at secondary recurrence, primary and secondary PFI, and the results were shown in Table 2. Variables significantly associated with PRS included PARPi history (Yes vs. No, HR=0.37, 95%CI 0.19-0.72, *p=*0.003) and secondary PFI (≥12 vs. 6-12 months, HR=0.47, 95%CI 0.23-0.94, *p=*0.034). In the multivariate analysis, the results showed that PARPi history remained significant (HR=0.46, 95%CI 0.23-0.89, *p=*0.022), whereas secondary PFI (HR=0.50, 95%CI 0.24-1.02, *p=*0.057) showed no statistical significance.

**Table 2:** The association of baseline factors with post-recurrence survival in patients treated with PARPi monotherapy and platinum-based chemotherapy after secondary relapse

	Univariable analysis		Multivariable analysis	
Characteristics	HR (95%CI)	*p* value	HR (95%CI)	*p* value
Age (years)				
≤49	0.70 (0.39-1.25)	0.223	--	--
>49	Reference		--	--
Regions of 3^rd^ line				
PARPi monotherapy	0.74 (0.41-1.35)	0.331	--	--
Chemotherapy	Reference		--	--
PARPi history				
Yes	0.37 (0.19-0.72)	**0.003**	0.46 (0.23-0.89)	**0.022**
No	Reference		Reference	
Residual Lesions		0.145		
R0	Reference		Reference	0.270
R1+R2	0.77 (0.39-1.54)	0.465	0.91 (0.46-1.82)	0.794
Unknown	0.45 (0.21-1.00)	**0.049**	0.53 (0.24-1.71)	0.115
BRCA mutation				
BRCA1	1.20 (0.58-2.49)	0.630	--	--
BRCA2	Reference		--	--
Stage at diagnosis				
I/II	0.72 (0.26-2.03)	0.534	--	--
III/IV	Reference		--	--
NAC-IDS				
Yes	0.82 (0.39-1.72)	0.600	--	--
No	Reference		--	--
1^st^ PFI (months)				
≥12	1.23 (0.63-2.37)	0.546	--	--
<12	Reference		--	--
2^nd^ PFI (months)				
≥12	0.47 (0.23-0.94)	**0.034**	0.50 (0.24-1.02)	0.057
<12	Reference		Reference	
CA-125 at secondary recurrence (U/ml)		0.211	--	--
≤70	Reference			
>70	0.70 (0.35-1.41)	0.316	--	--
Unknown	0.47 (0.20-1.09)	0.078	--	--

*PARP* Poly (ADP-ribose) polymerase (PARP) inhibitors, *BRCA* Breast cancer susceptibility gene, *NAC-IDS* Neoadjuvant chemotherapy and interval debulking surgery, *PFI* Platinum-free interval, *CA* Carbohydrate antigen

## Discussion

To our knowledge, this is the first study on the therapeutic effect of PARPi monotherapy compared with platinum-based chemotherapy, and the impact to subsequent platinum-based chemotherapy and survival after the resistance of PARPi in BRCA1/2-mutated patients with secondary platinum-sensitive relapse.

In patients with recurrent BRCA1/2-mutated ovarian cancer, PARPi monotherapy has been studied in several clinical trials, even though the indications have been recently withdrawn [8], and it has also achieved successful application in BRCA-deficient patients with prostate cancer and pancreatic cancer [18, 19]. In BRCA1/2 mutant patients with platinum-sensitive relapse who received at least two lines of platinum-based chemotherapy, the ORR ranged from 56.0% with niraparib to 80.0% with rucaparib [20–24]. Ovarian cancer patients with BRCA1/2 mutations were inherently more sensitive to platinum-based chemotherapy than patients with wild-type ovarian cancer [25], and the benefits of using PARPi inhibitors versus platinum-based chemotherapy at the same relapse stage were still uncertain. In our study, for patients with secondary platinum-sensitive relapse, the ORR was 77.5% and 80.0%, and the median PFS was 11.2 and 11.0 months, respectively. The therapeutic effect of PARPi monotherapy and platinum-based chemotherapy was similar.

The mechanisms of PARPi and platinum-based chemotherapy are both related to DNA damage repair, which mainly resulted from a variety of lesions affecting homologous recombination (HR), nonhomologous end joining (NHEJ) for double strand breaks, and mismatch repair (MMR), etc [26] , and the drug resistance mechanisms of PARPi include alterations in DNA damage repair, reactivation of HR, and replication fork protection [27]. Theoretically, PARPi resistance may lead to subsequent platinum-based chemotherapy resistance. In Joo Ern's study [28], BRCA1/2 mutation patients who received 3-11 lines of platinum-based chemotherapy before Olaparib were included. After Olaparib resistance, the ORR of platinum-based chemotherapy was 40% (19/48), and the median PFS was 22 weeks, suggesting that there was still a partial response to platinum-based chemotherapy after PARPi resistance. Another study found that both platinum and non-platinum chemotherapy had a response rate after resistance of PARPi maintenance therapy, with median PFS of 7.0 and 8.5 months, respectively [29]. In our study, the median PFS of platinum-based chemotherapy after PARPi resistance was 7.0 months, which was similar to previous studies, and it was noteworthy that the effectiveness of platinum-based chemotherapy after PARPi resistance was positively correlated with the duration of PARPi administration. These results verified, to some extent, that although platinum-based chemotherapy had cross-resistance with PARPi, the application of PARPi would not have negative impact on the efficacy of platinum-based chemotherapy in the posterior line. Besides, it is worth noting that with the development of next-generation sequencing technologies such as genomics, metabolomics, and especially proteomics, it is hoped that accurate choices can be provided for the treatment of PARPi-resistant patients [30].

PFS is currently the most widely used primary endpoint in clinical trials of PARP inhibitors, and most clinical studies have been conducted in recent years, so data on OS are still limited. Based on the limited OS data, the FDA withdrew Niraparib, as well as Olaparib and Rucaparib for ovarian cancer patients after three lines of treatment or more, following an increased risk of death [9, 10]. In our study, survival analysis confirmed that PARPi monotherapy significantly prolonged the survival of patients with BRCA1/2 mutations with secondary platinum-sensitive relapse, and patients who received PARPi treatment in the posterior lines also had a better prognosis than those without PARPi history, which has guiding significance for clinical decisions.

Factors affecting survival of ovarian cancer patients included tumor histology, FIGO stage, BRCA mutation status, ascites, and whether no residual lesions could be achieved after primary debulking surgery [31]. In a study with up to 10 years of follow-up, the initial survival advantage in patients with BRCA1/2 mutations may reflect a higher initial sensitivity to chemotherapy, but this response does not predict long-term survival, the strongest predictor of long-term survival was no residual lesions at resection, however, widespread treatment of PARPi was not involved in this study [32]. Our study demonstrated that treatment with PARPi was the independent factor affecting the prognosis of BRCA1/2 mutant patients, which in part reflects the superior efficacy of PARP inhibitors in this population.

To a certain extent, our research has significant advantages. The most significant limitation of our retrospective study was the limited number of patients. BRCA1/2 mutations account for less than 30% of ovarian cancer patients [33], and those who did not met the criteria for secondary platinum-sensitive relapse were excluded, as were those on maintenance therapy with PARPi or bevacizumab. In addition, it was difficult to collect treatment information after the progression of PARPi. Although the results of the analysis in our study were significantly different, further studies with large samples should be necessary. The findings of this study were applied only to a specific subset of the ovarian cancer patient population, not to all patients in general.

## Conclusion

For patients with BRCA1/2-mutated ovarian cancer with secondary platinum-sensitive recurrence, the therapeutic effect of PARPi monotherapy and platinum-based chemotherapy was similar. PARPi monotherapy does not negatively affect the efficacy of subsequent platinum-based chemotherapy after the progression of PARPi monotherapy, and could improve prognosis.

### Supplementary Information

**Additional file 1: **Baseline characteristics of patients treated with platinum-based chemotherapy after PARPi resistance in study group.

## Data Availability

All the original data of this study were available upon reasonable request to the corresponding author (songkun2001226@sdu.edu.cn), including, but not limited to, the request for repeating the results in this manuscript.
