# Supplementary material for: Efficacy of poly (ADP-ribose) polymerase inhibitors monotherapy and the impact to subsequent platinum-based chemotherapy in breast cancer susceptibility genes1/2-mutated ovarian cancer patients with secondary platinum-sensitive relapse
Source: J Ovarian Res. 2023 Oct 28;16:209. doi: 10.1186/s13048-023-01283-2 (PMC10612277; doi:10.1186/s13048-023-01283-2)
Supplement: Supplementary file 1 — Additional file 1: Baseline characteristics of patients treated with platinum-based chemotherapy after PARPi resistance in study group. [file 13048_2023_1283_MOESM1_ESM.docx]

**Additional file 1. Baseline characteristics of patients treated with platinum-based chemotherapy after PARPi resistance in study group.**

|  | **PARPi treatment**  **≥12 monhts**  **(n = 23)** | | **PARPi treatment**  <**12 monhts**  **(n = 28)** | | **p-value** | |  |
| --- | --- | --- | --- | --- | --- | --- | --- |
| Age at diagnosed (years) |  | |  | |  | |  |
| ≤49 years | 15 (65.2%) | | 19 (67.9%) | | 0.842 | |  |
| >49 years | 8 (34.8%) | | 9 (32.1%) | |  | |  |
| BRCA-germline-mutation status |  | |  | |  | |  |
| BRCA1 mutation | 15 (65.2%) | | 24 (85.7%) | | 0.086 | |  |
| BRCA2 mutation | 8 (34.8%) | | 4 (14.3%) | |  | |  |
| NAC-IDS |  | |  | |  | |  |
| Yes | 4 (17.4%) | | 5 (17.9%) | | 0.965 | |  |
| No | 19 (82.6%) | | 23 (82.1%) | |  | |  |
| FIGO stage at diagnosed |  | |  | |  | |  |
| I/II | 6 (26.1%) | | 2 (7.1%) | | 0.064 | |  |
| III/IV | 17 (73.9%) | | 26 (92.9%) | |  | |  |
| Primary tumor location |  | |  | |  | |  |
| Ovary | 23 (100.0%) | | 27 (96.4%) | | 0.360 | |  |
| Fallopian tube | 0 (0.0%) | | 1 (3.6%) | |  | |  |
| Histologic type |  | |  | |  | |  |
| High-grade serous | 21 (91.3%) | | 27 (96.4%) | | 0.193 | |  |
| Serous not specified | 0 (0.0%) | | 1 (3.6%) | |  | |  |
| Endometrioid | 2 (8.7%) | | 0 (%) | |  | |  |
| Residual lesions |  | |  | |  | |  |
| No | 7 (30.4%) | | 11 (39.3%) | | 0.492 | |  |
| Yes | 10 (43.5%) | | 10 (35.7%) | |  | |  |
| Unknown ^a^ | 6 (26.1%) | | 7 (25.0%) | |  | |  |
| PFI after 1^st^ line of platinum-containing chemotherapy |  | |  | |  | |  |
| <12 months | 8 (34.8%) | | 10 (35.7%) | | 0.945 | |  |
| ≥12 months | 15 (65.2%) | | 18 (64.3%) | |  | |  |
| PFI after 2^nd^ line of platinum-containing chemotherapy |  | |  | |  | |  |
| ≥6, <12 months | 16 (69.6%) | | 21 (75.0%) | | 0.665 | |  |
| ≥12 months | 7 (30.4%) | | 7 (25.0%) | |  | |  |
| CA-125 level at secondary platinum-sensitive relapse |  | |  | |  | |  |
| ≤70 U/ml | 6 (%) | | 7 (25.0%) | | 0.915 | |  |
| >70 U/ml | 16 (%) | | 20 (71.4%) | |  | |  |
| Unknown ^a^ | 1 (%) | | 1 (3.6%) | |  | |  |
| Tumor response of PARPi |  | |  | |  | |  |
| PR/CR | 21 (91.3%) | | 22 (78.6%) | | 0.213 | |  |
| SD/PD | 2 (8.7%) | | 6 (21.4%) | |  | |  |
| Hematological toxicity (≥ 3 CTCAE) | |  |  | | |  | |
| Yes | | 10 (43.5%) | 10 (35.7%) | | | 0.374 | |
| No | | 10 (43.5%) | 17 (60.7%) | | |  | |
| Unknown ^a^ | | 3 (13.0%) | 1 (3.6%) | | |  | |
| Chemotherapy regimens of 4^th^ line | |  | |  | |  | |
| Carboplatin based | | 12(52.2%) | | 16 (57.1%) | | -- | |
| Nedaplatin based | | 4 (17.4%) | | 4 (14.3%) | |  | |
| Cisplatin based | | 4 (17.4%) | | 1 (3.6%) | |  | |
| Oxaliplatin based | | 2 (8.7%) | | 2 (7.1%) | |  | |
| Lobaplatin based | | 0 (0.0%) | | 3 (10.7%) | |  | |
| Multiple platinum | | 1 ^b^ (4.3%) | | 2 ^c^ (7.1%) | |  | |

^a^ Data identified as unknown were not included in the difference analysis between the two groups.

^b^ Carboplatin+nedaplatin: 1 patient.

^c^ Carboplatin+cisplatin: 1 patient; Carboplatin+nedaplatin: 1 patient.

BRCA, breast cancer susceptibility gene; NAC-IDS, neoadjuvant chemotherapy and interval debulking surgery; FIGO, International Federation of Gynecology and Obstetrics; PFI, Platinum-free interval; CA, carbohydrate antigen; PARP, poly (ADP-ribose) polymerase (PARP) inhibitors; CTCAE, Common Terminology Criteria for Adverse Events.
